# Microleakage of chairside moulded, 3D-printed and milled provisional restorations using a curve-fit approach

**DOI:** 10.1590/0103-6440202305644

**Published:** 2023-12-22

**Authors:** Keshia Reyes, Polina Plaksina, Abdullah Barazanchi, Wendy-Ann Jansen van Vuuren, J Neil Waddell, Kai Chun Li

**Affiliations:** 1 Sir John Walsh Research Institute, University of Otago, Dunedin, New Zealand

**Keywords:** provisional, microleakage, 3D printing. Curve fit, image processing

## Abstract

The purpose of this study was to evaluate and measure the microleakage inhibiting quality of provisional restorations manufactured using computer-aided manufacturing, 3D printing, and chairside molded provisional restorative materials. Fifteen provisional restorations each from 3D printed, milled, and chairside molded were manufactured. All restorations were cemented onto sintered zirconia abutment dies and adhered with zinc-oxide non-eugenol temporary cement. Artificial aging was conducted by thermocycling for 800 cycles to simulate 1 month of clinical use. All specimens were submerged in 2% (w/w) methylene blue for 24 hours at 37°C, sectioned, and analyzed digitally for the distance of dye penetration through image analysis. The data were analyzed using the Kruskal-Wallis test with Dunn-Bonferroni post-hoc. Significant differences in dye penetration depth were observed between all groups except milled vs chairside molded. Light microscopy revealed differences in mean cement thickness for 3D printed, milled, and chairside molded of 83.6 µm (1σ = 31.9 µm), 149.1 µm (1σ = 88.7 µm) and 137.9 µm (1σ = 67.2 µm) respectively. Conclusion: 3D printed provisional restorations were found to have the least amount of microleakage compared to milled and chairside molded provisional restorations.

## Resumo

O objetivo deste estudo foi avaliar e medir a qualidade de inibição de microinfiltração de restaurações provisórias fabricadas usando manufatura assistida por computador, impressão 3D e materiais de restauração provisória moldados no consultório. Foram fabricadas 15 restaurações provisórias impressas em 3D, fresadas e moldadas em consultório. Todas as restaurações foram cimentadas em matrizes de pilar de zircônia sinterizada e aderidas com cimento temporário de óxido de zinco sem eugenol. O envelhecimento artificial foi conduzido por termociclagem por 800 ciclos para simular 1 mês de uso clínico. Todos os espécimes foram submersos em azul de metileno a 2% (p/p) por 24 horas a 37°C, seccionados e analisados digitalmente quanto à distância de penetração do corante por meio de análise de imagem. Os dados foram analisados usando o teste de Kruskal-Wallis com post-hoc de Dunn-Bonferroni. Foram observadas diferenças significativas na profundidade de penetração do corante entre todos os grupos, exceto entre fresado e moldado na cadeira. A microscopia óptica revelou diferenças na espessura média do cimento para as restaurações impressas em 3D, fresadas e moldadas em cadeira de 83,6 µm (1σ = 31,9 µm), 149,1 µm (1σ = 88,7 µm) e 137,9 µm (1σ = 67,2 µm), respectivamente. Conclusão: As restaurações provisórias impressas em 3D apresentaram a menor quantidade de microinfiltração em comparação com as restaurações provisórias fresadas e moldadas no consultório.

## Introduction

Provisional restorations are an essential step in fixed prosthodontic treatment. A high reduction of enamel and dentine is required for the provisional restoration to be cemented, pursuing sealed dentine tubules and marginal seals before the fabrication of the permanent restoration^1^. Failure to produce a clinically acceptable temporary restoration could expose the dentine tubules of the prepared tooth^1^ causing deterioration of the restoration; this could lead to damage to the surrounding tissues, poor aesthetics, and microleakage.

Microleakage occurs when there is a microscopic gap between the cement and tooth, allowing bacteria, ions, and molecules^2^ to seep in and adversely affect the tooth-cement interface. The consequences of microleakage include pulp inflammation and further necrosis, marginal discoloration, crown loosening of the provisional restoration, and post-operative sensitivity^2^. Some chairside provisional materials are prone to high water solubility and/or polymerization shrinkage, these factors alongside porosity included during the mixing process, can result in poor marginal adaptation and low flexural strength of the temporary restoration^3^, which further promotes microleakage. The exposure of temporary restorations to intra-oral temperature and pH fluctuations is unavoidable because of the food and drink consumed during the day or patient habits^4^. These factors can have a disadvantageous effect on provisional chair-side-made restorations and may compromise treatment^1^.

While provisional restorations were traditionally fabricated chairside through molding techniques, advances in technology have expanded options to allow fabrication using computer-aided design and manufacturing (CAD/CAM) and 3D printing. In general, CAD/CAM-manufactured provisional restorations allow the evaluation of aesthetics^5^, better protection of the pulp^2^, and can be more time efficient in an optimized digital workflow^6^.

CAD/CAM technology allows for two modalities of manufacturing dental devices, these are subtractive and additive manufacturing. Subtractive manufacturing, like computer numerically controlled (CNC) milling, can however be quite wasteful^7^, as the process involves grinding or cutting a pattern from a larger mass of material. The accuracy of milled provisional restorations also depends on milling bur tolerance, bur size, material properties, and the range of motion available from the milling unit itself.

Additive manufacturing such as 3D printing works by building up a 3-dimensional object layer-by-layer and has become a very popular and cost-effective way for producing dental devices. It is more economical than the milling process, as no material is wasted, and excess can be re-used for future fabrication. 3D printing using digital light processing (DLP) techniques, are also extremely time efficient, allowing the fabrication of a multitude of provisional restorations in a relatively short period. 3D printers are also more affordable compared to milling machines and accuracy can be controlled by the user via a range of settings^8^. However, due to the additive nature of 3D printing, adhesion between layers can be a potential issue, which can impede the strength of the material^9^.

Available literature suggests that provisional restorations fabricated through milling provide a superior fit as well as better strength, while 3D printed materials provide superior marginal accuracy, comparable strength, and a more affordable alternative than chairside restorations^10^ ^,^ ^11^. However, there is limited information available on the durability of the cement bond after artificial aging treatments such as thermocycling, and no standardization around quantitative analyses of marginal and internal fit^12^ ^,^ ^13^. Some studies^1^ ^,^ ^14^, that investigated microleakage have focused on qualitative approaches such as assessing the margin or dye penetration by a score-based assessment and using high-resolution microscopy to observe the marginal discrepancy or internal fit. Others^2^ ^,^ ^15^ have tried to measure the linear penetration depth, which is more informative but does not consider the natural curvature of the tooth. All these methods have limitations on the reproducibility of the data due to the subjective nature of the measurements based on individual experimental setups. Furthermore, few studies^10^ have compared the effect of different provisional materials on the microleakage, particularly after artificial aging. 

The purpose of this *in vitro* study was to quantitatively investigate the resistance to microleakage of provisional restorations fabricated using 3D printing, CNC milling, and conventional chairside molding after artificial aging. The null hypothesis of this study is that there will be no significant difference in penetration depth for the cemented provisional restorations between 3D printed, CNC milled, and conventional chairside molding.

## Materials and method

All the materials were prepared according to the manufacturer’s recommendations and the information on these materials is given in Box 1.


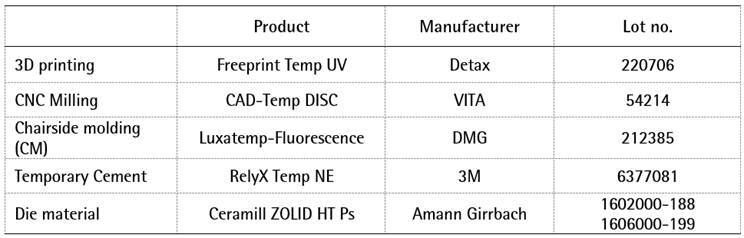


Box 1. Materials of the provisional restorations, cement, and dies

### Die design

Yttria-stabilized zirconia was selected for the die material due to the similarity in coefficient of thermal expansion with dentin and surface roughness finish^16^ ^,^ ^17^. To produce zirconia dies, a maxillary type IV die stone (HardRock, Shera, Germany) model was used to produce a standardized tooth preparation on the maxillary left 1^st^ molar, after which a polyvinyl siloxane (Exahiflex-Regular, GC, Japan) impression was taken to produce a working model. The PINDEX working model was prepared with Type IV stone following manufacturer instructions (Powder 100g: distill water 20ml), sectioned, and ditched according to conventional methods, before digitization using a digital benchtop 3D scanner (Ceramill Map 400, Amann Girrbach, Germany). The die scan was isolated and digitally altered to have a flat base and a total length of 15mm to ensure stability. Forty-five zirconia dies were milled (Ceramill Motion 2, Amann Girrbach, Germany) from the zirconia material and then sintered according to the manufacturer’s instruction for 11.5 hours at 1450°C (Ceramill Therm 3, Amann Girrbach, Germany). The zirconia tooth preparations were examined under an optical microscope for any imperfections or flaws.

### Fabrication of provisional restorations

A single unit standardized provisional restoration was designed on the maxillary left 1^st^ molar with a recommended 100-micron spacing^18^, using design software (Ceramill Mind, Amann Girrbach, Germany) and saved in a .STL file format. This design was used to fabricate identical restorations for all three specimen groups (3D printed, CNC Milled, and CM). Fifteen specimens were produced for each testing group. The 3D printed specimens (Freeprint Temp, DETAX) were made using a DLP 3D printer (Asiga Max, Asiga, Australia). After 3D printing, specimens were washed for 10 minutes in a fresh isopropyl alcohol bath to remove all traces of uncured resin after which, it was subjected to a final cure of 4000 flashes under N_2_ gas (Otoflash G171, Ivoclar Vivadent, Liechtenstein). The CNC-milled specimens were fabricated with a 5-axis milling unit (Ceramill Motion 2, Amann Girrbach, Germany) from CAD-Temp DISC. After milling, all connectors were removed and recontoured with a carbide bur.

The chairside molded (CM group) specimens were created by fabricating a silicone matrix (Protesil Labor, Vaninni Dental, Italy) using a previously fabricated 3D-printed specimen. The silicone matrix was then filled with chairside provisional material (Luxatemp-Fluorescence, DMG, USA) and placed on a zirconia die in the exact position marked by a notch and left to set as per manufacturer instructions. Excess material was removed around the margin using a scalpel blade. The margins of all 45 specimens were inspected for the presence of defects under light microscopy (SMZ800, Nikon, Japan).

### Cementing

Zinc-oxide non-eugenol cement (RelyX Temp NE, 3M, USA) was used according to the manufacturer’s instructions to bond each temporary restoration to a zirconia die under a load of 80N for 60s. The amount of pressure exerted during cementation was standardized and controlled using a universal testing machine (3369 Universal Testing Machine, Instron, USA). To maintain an evenly distributed load during cementation, a silicone matrix (Protesil Labor, Vaninni Dental, Italy) was created for the base of the die as well as the occlusal surface of the specimen.

### Thermocycling

After cementation, all prepared specimens were stored in distilled water in an incubator at 37°C for 24 hours. Specimens were artificially aged for 800 cycles to simulate approximately 1 month of clinical use^19^. This was accomplished by cycling specimens between 5°C and 55°C water baths, with a dwell time of 15s, using a thermocycler (Proto-tech, Dental Research Instruments).

To assess microleakage, a dye penetration approach was used. Acid-resistant red nail varnish (#003 Crimson Jelly, Revlon, USA) was used to coat the specimens approximately 1mm above the margin line to prevent the dye from penetrating through any cracks or imperfections other than the margin. All forty-five specimens were placed in a 37°C incubator (Incubator Labec, Australia) while submerged in 2% w/v methylene blue (Methylene blue C.I. 521015, Merck, Germany) for 24 hours; this enabled dye penetration at the cement-abutment and cement-restoration interfaces. All specimens were rinsed in distilled water until no trace of the dye was present on the external surfaces.

### Cross-sectioning

A customized 3D printed jig was fabricated to enable precise sectioning using a precision sectioning machine (Accustom-50, Struers, Denmark), of the specimens at the same position, this being perpendicular to the buccal surface and through to the palatal region. This was followed by image analysis.

### Image analysis

A digital camera (Sony A6400, Sony, Japan) mounted on an optical microscope (SMZ800, Nikon) was used to capture images at a resolution of 6000×4000 pixels with the sensor size of the camera set at 23.5mm ×15.6mm. Images were captured and analyzed from the margin beginning from the palatal region.

**Figure 1:**
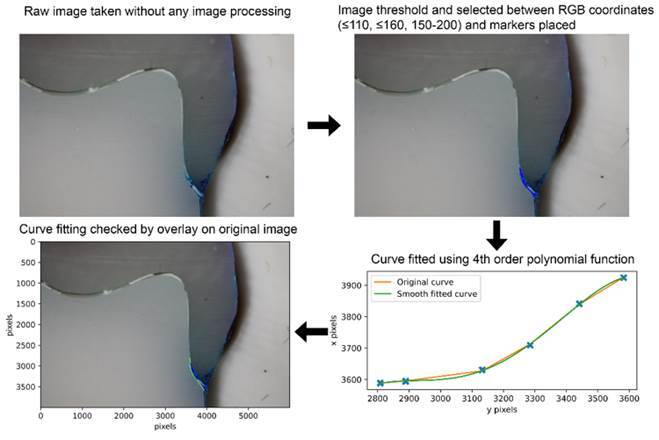
Step-by-step process of the image processing and curve fitting method used to determine the total dye penetrated distance. (A), raw image, (B). Image thresholding and placement of markers, (C). Curve fit of the actual line using a polynomial function (D). Overlay of the fitted curve on the original image for visual check.

A custom formula and software written in Python (Python 3.8.3 Software Foundation) were created for image analysis and measurement of dye penetration into the cement-tooth die interface, standardizing and yet simplifying the analysis process in a three-stage process. Firstly, dye penetration on the images was enhanced and selected according to a threshold of pixel colors using RGB coordinates (≤110, ≤160, 150-200). This was followed by placing green single-pixel (0, 255, 0) markers along the die-cement interface where the methylene blue penetrated the margin. Lastly, a second script would construct a curve of best fit and measure the penetration depth through the interface this enabled the study to quantify the true penetration depth of the dye as shown in Figure 1. The function used for the curve fitting was a 4^th^-order polynomial with the form:

$$y=ax^{4}+bx^{3}+cx^{2}+dx+e$$

Where a, b, c, d, e are the constants of the polynomial which control the shape of the curve. The length of the curve was determined using the arclength formula:

$$s=\int_{a}^{b}\sqrt{1+{\left(\frac{dy}{dx}\right)}^{2}dx}$$

Where a and b are the boundaries of the curve on the x-axis in pixels and dy/dx is the derivative of the polynomial function.

The pixel measurements were then converted to mm units by multiplying with a conversion factor of 0.0013028 mm/pixel, obtained by direct measurement using a microscope graticule. If the dye penetrated past the center of the die, then complete penetration is considered to have occurred and a maximum measurement of 7.18 mm (measurement from the margin to the center of the die) was assigned.

The cement thickness of the sectioned specimens was measured at three evenly spaced locations, starting from the margin and ending at the middle of the underlying occlusal surface.

### Statistical analysis

Power calculation (G*Power 3.1, Universität Düsseldorf, Germany) to estimate sample size was conducted using an effect size of 0.5 and statistical power of 0.8. The minimum sample size was determined to be 42, and therefore, a sample size of 45 was chosen. Shapiro-Wilk tests were used to verify the normality of the data. Kruskal-Wallis test with pairwise comparisons adjusted with Bonferroni’s correction was used to determine the statistical significance (p<0.05) for the microleakage dye penetration distance using statistical software SPSS v28. Mean and standard deviation was computed for both microleakage dye penetration distance and cement thickness.

## Results

**Figure 2:**
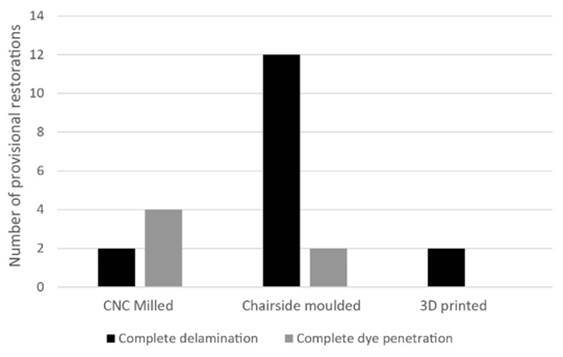
The amount of specimen failure/complete dye penetration within each group after artificial aging

Shapiro-Wilk tests for the 3 specimen groups (3D printed, CNC milled, and CM) were statistically significant (p<.05), therefore, the data significantly deviated from a normal distribution, and the Kruskal-Wallis test was used for statistical comparisons. The mean microleakage penetration distance after artificial aging for 3D printed, CNC milled, and CM was 2.20 mm (1σ = 2.15 mm); 4.68 mm (1σ = 2.44 mm) and 6.84 mm (1σ = 1.31 mm) respectively. Statistical significance was observed between 3D printed vs CNC milled (p=0.041) and 3D printed vs CM (p<0.0001) while no statistical significance was observed between CNC milled vs CM (p=0.092). Some of the provisional restorations were completely delaminated after artificial aging and the number of complete delamination and dye-penetrated provisional restorations for each specimen group is shown in Figure 2.

**Figure 3:**
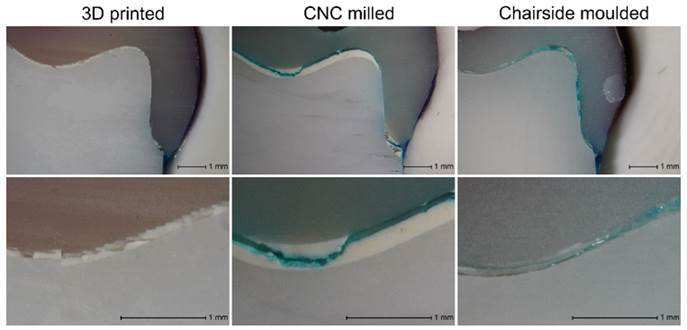
Light microscopy images showing the penetration of the dye, variation in cement thickness between materials, and differences in surface texture of the (A). 3D printed, (B). CNC milled, and (C). CM provisional restorations

The 3D-printed provisional restoration group showed the least amount of microleakage compared to CNC milled and CM groups (Figure 3). The cement thickness and surface texture between the three groups were also noticeably different. The average cement thickness for 3D printed, CNC milled and CM were 83.6 µm (1σ = 31.9 µm), 149.1 µm (1σ = 88.7 µm) and 137.9 µm (1σ = 67.2 µm). The CNC milled and CM groups showed inconsistent cement thicknesses but smooth interfaces overall, while the 3D printed group showed consistent cement thicknesses and distinctive textured interface caused by the additive layering inherent in 3D printing.

## Discussion

A key function of a provisional restoration is to protect the exposed tooth surface from the diffusion of bacteria and oral fluids for the interim period before a fixed restoration is in place. This period varies depending on the complexity of the treatment and unexpected circumstances causing further delays. Therefore, the provisional material must offer a leak-proof seal to protect the abutment tooth until a fixed restoration is completed^1^ ^,^ ^2^ ^,^ ^10^. The results from our study showed significant differences in dye penetration between CNC, CM, and 3D-printed provisional restorations. Therefore, we reject our null hypothesis that there would be no difference in dye penetration between the three provisional materials.

Ideally, the best option to imitate intra-oral conditions is to quantify microleakage using human or bovine teeth. However, standardization is an issue when working with real teeth and the focus of the current study was to use an objective approach to accurately quantify the degree of microleakage. Therefore, in this study, milled zirconia dies with a coefficient of thermal expansion (CTE) of 10.4±0.5×10^-6^K^-1^ was selected to mimic natural human dentin/enamel, which has a coefficient of thermal expansion range of (8.3-11.4)×10^-6^K^-1(^ ^20^. Since the accelerated aging process with thermocycling induces stresses by thermal contraction and expansion, zirconia is a good simulant material for dentin and enamel, although nevertheless, is still a limitation of the study. Furthermore, the surface roughness of milled zirconia is also within the range of typical tooth preparation by rotary instruments^16^.

Results from the microleakage test after 800 thermocycles, which simulates 1 month of clinical use, observed that CM had the worst outcome for dye penetration with a mean value of (6.84mm), followed by the CNC milled group (4.68mm), while the 3D printed group showed the lowest mean dye penetration of 2.20mm. As shown in Figure 2, 12 out of 15 restorations in the CM group failed by complete delamination of the restoration from the die after artificial aging, while the other 3 restorations which did not delaminate, observed partial to full penetration of the dye. The CNC milled restorations observed 2 out of 15 specimens that had completely delaminated, and 4 specimens exhibited complete dye penetration. The 3D printed restorations had 2 out of 15 restorations completely delaminate and none of the other restorations in the group had complete dye penetration. This seems to indicate that both the CM and CNC milled restorations were not as reliable as 3D printed restorations over a simulated 1-month of clinical use.

Several factors have been identified that could explain these observations. Firstly, roughness and higher surface area of the fitting surface of the restoration have been shown to influence its mechanical retention^21^. In this study, 3D printed restorations had a noticeably different internal surface texture compared to CNC milled and CM. The additive manufacturing method of 3D printed provisional restorations created a microscopic “stepped” surface as shown in Figure 3, resulting in a staircase-like internal surface. This greatly increased the total surface area for bonding and, therefore, likely led to better bonding between the cement and restoration. This also had a positive effect on the bonding between the zirconia and to cement interface, as shown by the low dye penetration and delamination failure in the 3D printed group. In the CNC milled and CM group, the internal surface of the restoration was smooth as shown in Figure 3, which resulted in a smaller bonded surface area, and weaker bond compared to the 3D printed provisional restorations.

Secondly, the cement layer and internal fit of the 3D-printed restorations were more consistent than CM and CNC-milled restorations. In CM restorations, the conventional technique does not account for a consistent cement spacing during fabrication, resulting in the uneven and thicker (µ = 137.9 µm, 1σ = 67.2 µm) cement layer observed. The CNC milled restorations in our study also had noticeable variations in the cement thickness (µ = 149.1 µm, 1σ = 88.7 µm), often thin around the axial walls, resulting in a thicker and uneven cement layer on the occlusal surface. The cause of this is likely due to inaccuracies from the milling process, even though new burs with manufacturer-recommended settings were used. This is also in agreement with Lee et al’s findings^22^ where the internal fit of milled restorations was significantly worse than 3D printed restorations, which is likely caused by the offset setting applied when the milling process is determined for manufacturing. The milling process also has limited capabilities to mill areas smaller than the diameter of the bur, thus the internal fitting surface is less accurate than those of 3D printed surfaces. This is also consistent with our findings, where our 3D printed restorations were closest to our designed cement thickness of 100 µm, with a mean of 83.6 µm and a standard deviation of 31.9 µm.

A previous study by Khng et al.^5^ also observed that milled provisional restorations had over 40% more microleakage than conventionally made restorations. This was concluded to be a result of the ideal cement spacing made with the restoration to be too small to be seated on the die, so it had to have a greater than-ideal cement layer^5^. In contrast, our study observed no significant difference between the CNC milled restorations and the conventional CM restorations in terms of microleakage dye penetration. All restorations in our study were seated using a consistent static force of 80N for 60 seconds. This was considered an achievable load that could be applied in a clinical situation using typical bite forces^23^ and hand pressure to fully seat the provisional restorations to the designed cement spacing, although statistical significance was not analyzed for the cement thickness between materials due to the uneven specimens groups from complete delaminated specimens after artificial aging.

Since there is no standard method of measuring microleakage, a limitation of our study is that it is difficult to compare our results with previous studies. Additionally, to the authors’ knowledge, no study has compared 3D printed, CNC milled, and chairside provisional restorations after artificial aging. The lack of standardized methods has resulted in many investigators resorting to developing their approach, based on clinical settings. One widely used approach to measure microleakage through dye penetration is the non-parametric scoring method^1^ ^,^ ^14^. This method assesses the degree of dye penetration with an integer score, usually between 0 to 5 where the higher the score, the higher the observed dye penetration. While simple to conduct, this approach is a subjective determination of microleakage as it does not measure the distance of the dye penetration, but simply looks at the length of the dye penetration along the axial wall. The present study aimed to improve on this method by using an objective approach to measure the exact amount of the dye penetration along the axial wall in millimeters through a series of image processing and curve fitting. The methodology developed here was able to accurately locate and measure the penetrated dye, producing a reasonably fitted curve as seen in Figure 2. Although subjectivity from non-parametric scoring methods does raise the question of measurement accuracy and error, one benefit from a scoring-based method is that it allows better comparison between different tooth sizes since the scores are based on the ratio of the axial wall to the occlusal surface. However, one can argue that this can be easily converted from direct measurements by measuring the distance from the margin to the occlusal surface and expressing the microleakage measurement as a ratio of this.

Another variable, not well standardized in literature, is the use of thermocycling to simulate the thermal cyclic conditions in the oral environment. Available literature has shown varying parameters of several thermocycles and dwell times, with the only consistent parameter being the two cycled temperatures of 5ºC and 55 ºC. In general, some studies used thermocycles between 250 and 1000 cycles^1^ ^,^ ^24^ but did not indicate what the number of cycles equates to in terms of months or years. Furthermore, some studies have justified that dye penetration in restored teeth did not significantly vary for cycles between 250-1500^25^ ^,^ ^26^. This, however, did not seem to apply to our study as the high number of complete delamination in the CM group, indicated that there would be significant differences in dye penetration at a lower number of thermocycles and cannot be generalized across all materials. As provisional restorations are not usually used for longer than a month, this study used the conservative estimate by Gale et al.^19^ of approximately 10,000 cycles for one year of clinical use, which equates to approximately 800 cycles to simulate one month of clinical use.

To determine the amount of dye penetration, the 4th-degree polynomial function showed a good fit when overlaid for visual inspection along the actual cement interface (Figure 2). This meant the method of analyzing the penetration depth was consistent and was a valid method to determine the severity of microleakage. Methylene blue worked well as a dye penetrant in the development of methodology for this study, but the manual sectioning severely limited the practicality of analyzing more cross-sections of each tooth. A potential solution would be to use silver nitrate solution, an alternative dye penetration agent, used by several microleakage studies^2^ ^,^ ^27^. The microleakage can then be assessed using X-ray microtomography and the volume of the dye penetration is determined by image thresholding. A similar analysis of the penetration distance could be applied to such an approach by thresholding the dye penetration and placing markers on multiple digital slices, which make up the 3D volume data. Applying this analysis to x-ray microtomography data will give much higher statistical reliability and throughput, since each digital slice is a cross-section of the tooth, making even analyzing an entire tooth simple and quick. However, this method of assessment may only be suitable for tooth foundations with large differences in the image contrast compared to silver, such as enamel/dentin. The present study serves as an important foothold toward the standardization of an easy and accurate method to quantify microleakage applicable to multiple assessment techniques. Future investigations should consider applying the present methods using X-ray microtomography data and computing the ratio of the dye penetration distance to the total distance to the occlusal surface for crowns cemented on different tooth sizes.

In conclusion, the results from this study observed that the 3D printed provisional restoration was least susceptible to microleakage after artificial aging, while chairside molded provisional restorations showed the highest risk of microleakage susceptibility. Therefore, within the limitations of this study, 3D-printed provisional material is the optimal material for high microleakage resistance and clinical scenarios where long-term provisional placement is required.
